# The art of strain improvement of industrial lactic acid bacteria without the use of recombinant DNA technology

**DOI:** 10.1186/1475-2859-13-S1-S5

**Published:** 2014-08-29

**Authors:** Patrick MF Derkx, Thomas Janzen, Kim I Sørensen, Jeffrey E Christensen, Birgitte Stuer-Lauridsen, Eric Johansen

**Affiliations:** 1 Innovation, Chr Hansen A/S, 10-12 Bøge Allé, DK2970, Hørsholm, Denmark

## Abstract

The food industry is constantly striving to develop new products to fulfil the ever changing demands of consumers and the strict requirements of regulatory agencies. For foods based on microbial fermentation, this pushes the boundaries of microbial performance and requires the constant development of new starter cultures with novel properties. Since the use of ingredients in the food industry is tightly regulated and under close scrutiny by consumers, the use of recombinant DNA technology to improve microbial performance is currently not an option. As a result, the focus for improving strains for microbial fermentation is on classical strain improvement methods. Here we review the use of these techniques to improve the functionality of lactic acid bacteria starter cultures for application in industrial-scale food production. Methods will be described for improving the bacteriophage resistance of specific strains, improving their texture forming ability, increasing their tolerance to stress and modulating both the amount and identity of acids produced during fermentation. In addition, approaches to eliminating undesirable properties will be described. Techniques include random mutagenesis, directed evolution and dominant selection schemes.

## Introduction

Lactic acid bacteria (LABs) are industrially important organisms used for the production of dairy products like yoghurt, cheese, buttermilk and kefir. Apart from fermenting milk, LABs are also used to ferment vegetables, meat, fish and cereals. Finally, LABs play an important role in the production of alcoholic beverages [1]. Species used for these applications typically belong to the genera *Lactococcus, Streptococcus, Pediococcus, Oenococccus, Leuconostoc *or *Lactobacillus *and have been isolated from natural habitats like plants, or fermented foods like dairy and meat products [2,3]. Apart from having a preservative effect by inhibiting the growth of spoilage microorganisms through the production of organic acids and consumption of nutrients, LABs also improve organoleptic properties of the product by producing metabolites that can enhance taste and texture [2]. The global dairy industry is constantly exploring new ways to improve products to fulfil consumers' demand for improved taste and texture or the reduction of additives, sugar, fat or overall calorie content. This pushes the boundaries of microbial performance and requires the constant development of new starter cultures with novel properties.

Progress made in the last decade in the field of laboratory automation and high throughput screening of microorganisms has significantly reduced the effort required to screen large collections for specific traits [4]. Wild type strains may have properties unique to the industry, but to fully exploit their potential, specific improvements are often required. In other cases it might be needed to reduce or eliminate an unwanted property. In addition, it can be of interest to improve strains which already have established industrial applicability. Recombinant DNA technology, due to its precision and versatility, would be an ideal technology to use to improve microbial performance were it not for restrictive food legislation and consumer acceptance issues for genetically modified food ingredients. Thus, all efforts to improve strains for industrial application are, today, based on natural strategies for strain improvement (*i.e*. without the use of recombinant DNA technology) such as random mutagenesis, directed evolution and dominant selection.

Random mutagenesis (classical strain improvement) has been used extensively in the food industry [5,6]. This approach is based on the introduction of random mutations into the genome of interest, characterization of a large subset of variants, and selection of strains with the desired property for further use. Despite many successes, the method is generally hampered by the fact that, apart from the desired mutation, many unintended mutations which could have a negative impact on performance are introduced [7].

Directed evolution (or adaptive evolution) is a technique in which a strain is slowly adapted to certain growth conditions reflecting an application parameter [8,9]. In this case the population is enriched for strains with the desired property but, here too, there is a risk of accumulation of unintended mutations (reviewed by Barrick and Lenski [10]).

Dominant selection is based on designing a selection scheme in which only strains with the desired property can grow [11]. Success of such a method requires considerable insight into microbial physiology. If the selection is powerful enough, strains with single mutations can be obtained without using mutagenic agents.

Furthermore, natural mechanisms like bacteriophage transduction, natural competence and conjugation can be mentioned as additional useful approaches since these are specifically excluded from the European Union's definition of recombinant DNA techniques provided that none of the strains involved are genetically modified organisms [12].

**Figure 1:**
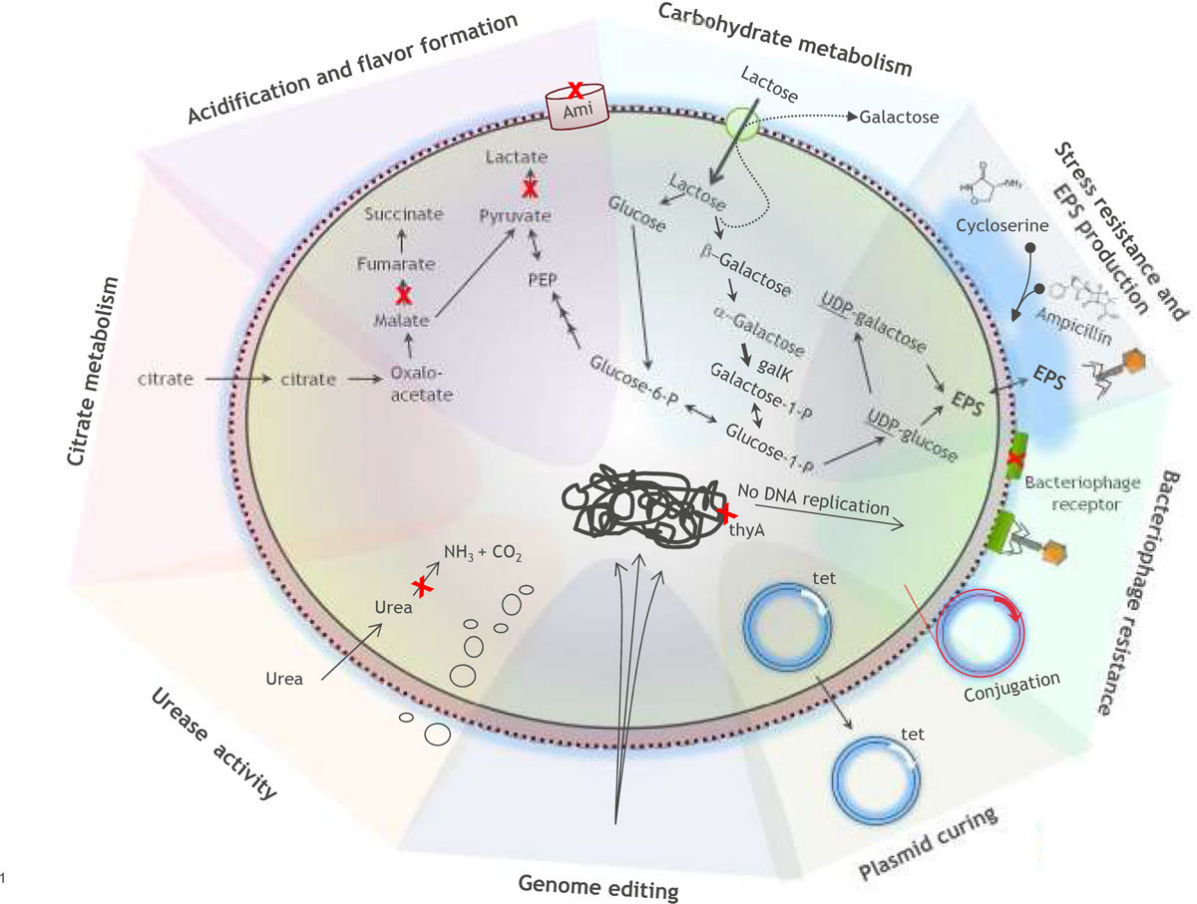
**Schematic representation of various classical strain improvement targets of LABs**. A cross (X) highlights the position of mutations or effect of mutations as described in the text.

In this review we will illustrate the use of the abovementioned methods with examples from the literature and our own laboratories and elaborate on the importance of natural strain improvement techniques (see Table 1 & Figure 1 for an overview). Focus will be on *Lactococcus, Streptococcus *and *Lactobacillus *primarily used for dairy fermentations.

**Table 1:** Overview of methods described in this review.

Method used	Advantages	Disadvantages	Topic	Aim
**Random mutagenesis**	Little knowledge required. Especially useful for elimination of specific characteristics where direct selection is not possible.	Use of dangerous chemicals. Mutation bias and hot spots. Second site mutations will be created. Requires screening of a large number of survivors.	Pyrimidine auxotrophy	Bacteriophage resistance
			
			Elimination of antibiotic resistance	Eliminating unwanted property
			
			Elimination of citrate metabolism	Eliminating unwanted property
			
			Urease negative mutants of *S. thermophilus*	Eliminating unwanted property
			
			*Lactobacillus *strains with low post acidification	Improving product stability

**Directed evolution**	Little knowledge required. Especially useful for complex phenotypes. Mutagens normally not required.	Multiple mutations may occur. May require complex experimental setups. Slow process requiring long time frames. Selection is at population level, isolated strains must be characterized.	Increasing the growth yield during fermentation	Increased efficiency in culture production

**Dominant selection**	Mutagens not required. Often results in a single mutation.	Requires considerable insight into physiology of the cell. Analogues may be toxic to humans.	Bacteriophage receptors	Bacteriophage resistance
			
			Conjugation	Bacteriophage resistance
			
			Modifying bacterial cell surfaces	Improving texture
			
			Optimizing the metabolic pathway of EPS	Improving texture
			
			Bacteriophage resistant mutants	Improving texture
			
			*Lactobacillus *strains with improved ethanol or bile tolerance	Improving survival and efficacy
			
			*Lb. helveticus *producing succinate	Improving flavor
			
			Altering acidification properties by adapting the carbohydrate metabolism	Overcoming effect of a mutation

**Recombinant DNA technology**	Extremely accurate targeted methods.	Consumer acceptance. Regulatory approval. Can be difficult to apply to industrial strains.	Potentially all of the above	Potentially all of the above

## Improving bacteriophage resistance

The prevention of bacteriophage attack is one of the most important challenges in dairy fermentations. Although naturally occurring bacteriophage resistance mechanisms are present in LABs, it is necessary to continuously improve bacteriophage resistance due to the high adaptability and diversity of bacteriophages [13,14]. Many bacteriophage infections can be prevented by using a culture replacement or rotation system based on LABs belonging to different bacteriophage sensitivity groups. Here we focus on *Lactococcus lactis *due to their importance and high frequency of use in dairy fermentations. Several approaches for making bacteriophage resistant mutants of *L. lactis *are described below. These include refinement of traditional methods like the isolation of spontaneous bacteriophage resistant mutants, conjugative transfer of bacteriophage resistance plasmids, as well as a novel approach based on understanding the basic biology of bacteriophage infection.

### Bacteriophage receptors

The *Lactococcus *PIP protein (**P**hage **I**nfection **P**rotein) [15], encoded by the *pip *gene, is the receptor for bacteriophages of the prolate-headed c2 species and was believed to be used by all c2 bacteriophages [16]. While generating c2-resistant mutants of industrial strains of *L. lactis *using the pGhost9:ISS1 integration system [17] we found bacteriophage resistant mutants which did not contain an ISS1 integration in the *pip *gene. Instead, the integration was found to be in the *yjaE *gene, encoding a protein of unknown function [18]. The *yjaE *gene shows only 22% identity to the *pip *gene and is predicted to encode a putative ABC-2-like protein with six membrane-spanning regions with N- & C-terminal phage infection protein domains (IPR017500 and IPR17501) and several extended heptad repeats (TIGR03057). Although *yjaE *has a domain with high similarity to the *pip *phage infection domain, the low degree of identity makes it clear that YjaE is a unique cellular component. Generation of gene disruption mutants confirmed that inactivation of the *yjaE *gene renders strains completely resistant to a number of bacteriophages of the c2 species as well as to two bacteriophages of the 936 species (Table 2). Bacteriophages that require YjaE for infection (CHPC3, CHPC24, CHL92, bil67) are not affected by disruption of the *pip *gene, and bacteriophages which require the PIP protein (CHPC180, c2) can still infect *yjaE *mutants. Thus, the two best characterized type c2 bacteriophages, c2 and bIL67 [19], use different receptors: c2 uses PIP and bIL67 uses YjaE.

**Table 2:** Bacteriophage sensitivity of *yjaE *and *pip *mutants.

	Phage species	IL1403	IL1403 ΔyjaE GMO	IL1403 ΔyjaE spont.	IL1403 Δpip GMO	CHCC 7552	CHCC 7552 ΔyjaE GMO	CHCC 7552 ΔyjaE **spont**.
**CHPC3**	c2	+	-	-	+	+	-	-
**CHPC24**	c2	+	-	-	+	+	-	-
**CHPC180**	c2	+	+	+	-	-	-	-
**CHL92**	c2	+	-	-	+	+	-	-
**bIL67**	c2	+	-	-	+	-	-	-
**c2 **	c2	+	+	+	-	-	-	-
**CHPC234**	936	-	-	-	-	+	-	-

Legend table 2:

+ : lysis by bacteriophage; - : no lysis by bacteriophage

GMO: produced with recombinant DNA technology

spont.: spontaneous mutant generated by challenge with bacteriophage requiring YjaE (*e.g*. CHPC24).

We isolated spontaneous bacteriophage resistant mutants of five different industrial *L. lactis *strains by challenging with different bacteriophages using the YjaE-receptor and found that all resistant mutants investigated had mutations within the *yjaE *gene. Out of 21 bacteriophage resistant mutants, 18 strains had single nucleotide changes generating premature stop codons upstream of the membrane-spanning regions and resulting in a truncated protein. The three remaining mutants had deletions within the membrane anchoring region, the promoter region or the external loop. To date, we have not observed bacteriophage using the YjaE-receptor being able to overcome the resistance of YjaE deficient strains. Importantly, the inactivation of the *yjaE *gene does not affect the acidification profile of industrial *L. lactis *strains. This method is thus ideal for generating new bacteriophage resistant starter cultures without compromising the performance of the culture during application [18].

### Conjugation

Improvement of bacteriophage resistance by conjugation is frequently described in the literature, especially for *L. lactis*, where many bacteriophage resistance systems are localized on plasmids [20]. The bacteriophage resistance of *L. lactis *CHCC1915 and CHCC1916 was improved by conjugative transfer of the bacteriophage resistance plasmid pCI1750 from *L. lactis *UC653 harboring the resistance system AbiG [21]. This abortive infection (Abi) mechanism is encoded by two genes, *abiGi *and *abiGii*, conferring resistance to bacteriophages of the 936 species, and partial resistance to c2 species bacteriophages [22]. For mating experiments, a lactose negative derivative (MG1363 containing pCI1750) was used as plasmid donor, and transconjugants were selected by challenging with bacteriophages inhibiting the recipient followed by plating on lactose indicator agar plates. Transconjugants of CHCC1915 and CHCC1916 have been on the market for more than 20 years and are still performing well, although several bacteriophage have been isolated which are unaffected by the AbiG resistance system.

### Pyrimidine auxotrophy

In contrast to the previous examples where bacteriophage resistance is not established for every bacteriophage attacking a certain strain, it is possible to develop completely resistant mutants by abolishing bacteriophage DNA replication [23,24]. Thymidylate synthase, encoded by the *thyA *gene, is essential for the *de novo *synthesis of dTTP. Since milk is devoid of thymidine, replication of DNA, including DNA from an infecting bacteriophage, is abolished in *thyA *mutants. MBP71, a *thyA *mutant of *L. lactis *CHCC373 with a 42-bp deletion at the beginning of the *thyA *gene shows complete resistance towards nine bacteriophages from the 936 and P335 species. Because *thyA *mutants are still able to synthesize RNA and thereby also proteins, mutants are still metabolically active. Since cell division does not occur, it is necessary to increase the inoculation rate to reach an acidification activity similar to that of the wild type strain [23]. MBP71 was created using recombinant DNA technology and exclusively used for proof-of-concept. Mutagenesis and screening for pyrimidine auxotrophy has subsequently been used to obtain variants of industrial strains of *L. lactis *and *S. thermophilus *suitable for inclusion in starter cultures. No bacteriophage variants that overcome this resistance mechanism have been discovered to date.

## Improving texture of fermented milk

Pectins and starch are often used to create the desired texture in fermented milk products. These additives can, however, be rendered unnecessary through development of LABs which create the desired texture. Especially the production of extracellular polysaccharides (EPS) has been in focus since EPS is known to contribute to the texture of fermented milks [25]. Here we describe several natural strain improvement methods to improve the ability of LABs to texturize fermented milk.

### Modifying bacterial cell surfaces

The bacterial cell surface is in direct contact with the external environment and is expected to be involved in binding of environmental components and formation of texture in milk during fermentation. EPS production has previously been found to be highly strain dependent and strongly correlated to the *Streptococcus thermophilus *genotype and especially to the genetic content of the *eps *gene cluster [26]. Texture formation is, apart from EPS [27], also affected by other bacterial cell surface components [28,29]. The structure and function of the cell surface of lactic acid bacteria has recently been reviewed [30].

To further exploit the role of the cell surface in the formation of texture in fermented milk, we applied a dominant selection strategy to isolate mutants with cell surface alterations. Selection was based on resistance to compounds interfering with the biosynthesis of cell surface components; *viz*. D-cycloserine (D-4-amino-isoxazolidone) and ampicillin. D-cycloserine is an antibiotic which inhibits enzymes involved in D-alanine metabolism and can cause cell lysis [31,32]. Ampicillin is an antibiotic which inhibits the transpeptidase responsible for crosslinking peptidoglycan and can also cause cell lysis [33]. Mutants of *Lactobacillus delbrueckii *subsp. *bulgaricus *which are resistant to either D-cycloserine or ampicillin are readily obtained. Some of these show reduced whey syneresis after acidification of milk and improved texturizing properties as determined by rheology measurement [14]. Preliminary experiments show that these mutants have changes in their cell surfaces which affect their interactions with the external environment.

### Optimizing the metabolic pathway of EPS

Most strains of *S. thermophilus *are unable to grow on galactose as sole carbon source in spite of the presence of intact genes encoding the required enzymes. Increased EPS production in *S. thermophilus *was observed following enhancement of the activity of enzymes in the galactose metabolic pathway (e.g. phosphoglucomutase and glucose-1-phosphate uridylyltransferase) by genetic manipulation [34,35]. Also, production of EPS by ropy *S. thermophilus *strains was increased through genetically targeted enhancement of galactokinase activity. However, combining such a mutant with a *Lb. delbrueckii *subsp. *bulgaricus *strain for yoghurt production did not result in significant overproduction of EPS, and the texturizing properties of the yoghurt were not improved [36]. Improvement of the rheological parameters of yoghurt by modifying enzymatic activity in the galactose metabolic pathway without the use of recombinant DNA techniques has not been reported.

*S. thermophilus *CHCC6008 is used extensively in the dairy industry because of its ability to texturize fermented milk. Strain CHCC11379 was isolated as a spontaneous galactose positive mutant of CHCC6008 as described in the section describing dominant selection. The ability of CHCC11379 to texturize fermented milk was increased by 10%, measured as shear stress, compared to its parent CHCC6008 [28]. Analysis of the galactose operon of CHCC11379 revealed a G to A mutation within the -10 position of the *galK *promoter resulting in a perfect "TATAAT" Pribnow box [37]. This optimized Pribnow box increased transcription of the genes from the galactose operon (*galK, galT, galE, and galM*) 2.5 to 3.7-fold. The amount of excreted EPS during milk fermentation was, however, not significantly increased for CHCC11379 suggesting that the ability to ferment galactose leads to a change in composition of the EPS rather than to an increase in the quantity. In contrast to the results from Robitaille *et al*. [36], viscosity and shear stress were increased when CHCC11379 was used in a yoghurt culture together with an EPS positive *Lb. delbrueckii *ssp. *bulgaricus *strain. This indicates that texture in milk is not determined solely by the amount of excreted EPS; EPS structure and the interaction between different EPS types also play a role.

### Bacteriophage resistant mutants

Capsular polysaccharides (CPS) can provide a protective barrier against bacteriophage infection although it was reported that it was not possible, so far, to increase EPS production using bacteriophages as selective agent [38]. Nevertheless, we speculated that selecting for bacteriophage resistance in CPS positive and, at the same time, EPS excreting *S. thermophilus *strains could result in mutants with increased CPS production and improved texture. *S. thermophilus *CHCC11977 was isolated as a bacteriophage-resistant mutant of a galactose-positive *S. thermophilus *strain, and its texturizing properties in milk, measured as shear stress and viscosity, were improved by 20% compared to its parent [39]. The amount of excreted EPS was also increased by 20% in CHCC11977.

## Removal of undesirable traits

LABs are used in food fermentations due to their many desirable properties. However, certain properties may be undesired in the food chain while others might be undesirable under certain conditions. Elimination of an undesirable property will be an improvement of the industrial properties of a strain.

### Elimination of antibiotic resistance

The increasing resistance of pathogenic bacteria to commonly used antibiotics is a growing threat to public health [40]. Resistance can either be acquired by mutation or by the transfer of antibiotic resistance genes from other bacteria. Bacteria intentionally added to the food supply should not contain transmissible antibiotic resistance genes. For this reason, all strains which are intended for use in the food chain are tested genotypically [41] and phenotypically [4] for antibiotic resistances not normally observed in the relevant species. Strains with atypical patterns are further characterized and those with potentially transmissible antibiotic resistance genes are either eliminated from further product development or measures are taken to eliminate or inactivate the undesired antibiotic resistance determinant.

Transmissible antibiotic resistance genes are often located on transposable elements or plasmids. While this increases the likelihood of transfer, it also simplifies the elimination process since these elements are often genetically unstable.

*Lb. crispatus *CHCC3692 contains an *erm*(B) gene encoding high level resistance to erythromycin on a 3.2 kb transposon. Heat shock treatment at 60°C was found to induce the expression of a transposase and resulted in excision of *erm*(B) from the chromosome [42].

Plasmids encoding antibiotic resistance genes are common in lactic acid bacteria and especially in *Lactobacillus *isolates. A number of different methods exist for elimination of a plasmid from a strain; the optimal method being highly strain dependent [43]. Acridine orange and mitomycin-C were used to eliminate an *erm *gene from a strain of *Lb. fermentum *[44] while novobiocin was used to eliminate a *tet*(M) and a *tet*(S) gene, respectively, from two different strains of *Lb. plantarum *[45,46]. Protoplast formation and regeneration was the only method found suitable to remove two plasmids encoding tetracycline resistance (*tet *(W)) and lincosamide resistance (*lnu*(A)) from a commercially used probiotic *Lb. reuteri *strain [47].

In some cases, it might be of interest to eliminate or inactivate antibiotic resistance genes which have not been shown to be transmissible and which do not reside on a transposon or plasmid. An example of this is the *tet*(W) gene responsible for tetracycline resistance in all known members of *Bifidobacterium animalis *subsp. *lactis *[48]. Ultraviolet light mutagenesis of cells grown in the presence of ethidium bromide was used to obtain tetracycline-sensitive mutants of strains bearing a chromosomal *tet*(W) gene. For all mutants tested, it was possible to obtain strains with mutations in the *tet*(W) gene [48,49].

### Elimination of citrate metabolism

The ability to catabolize citrate to acetate, carbon dioxide and pyruvate is present in many LABs, and citrate utilization is often associated with the production of acetoin and diacetyl [50]. While these compounds improve the properties of some fermented products (e.g. buttermilk and some cheeses), excessive production is unwanted in others (e.g. wine and cottage cheese).

During malolactic fermentation in wine by *Oenococcus oeni*, the accumulation of acetate and diacetyl resulting from citrate metabolism can negatively impact the sensory properties [51]. Random mutagenesis was used to eliminate citrate utilization in a commercial *O. oeni *strain. The resulting variant contains a nonsense mutation in the citrate transporter gene [4]. The ability to utilize citrate in *O. oeni *is plasmid encoded [52] suggesting plasmid curing as an alternative approach as described in the section describing random mutagenesis.

The ability of *L. lactis *subsp. *lactis *biovar *diacetylactis *to utilize citrate is also plasmid encoded [53]. This feature is important for the production of flavor compounds and carbon dioxide in cheeses like Danbo, Havarti and Gouda, but these compounds are unwanted for the production of cottage cheese (see also the section describing random mutagenesis). Elimination of the citrate plasmid in *L. lactis *subsp. *lactis *biovar *diacetylactis *can be achieved by heat-shock or treatment with novobiocin resulting in strains useful for cottage cheese production [54].

### Urease negative mutants of *S. thermophilus*

Traditionally, cottage cheese is made by fermenting milk with *L. lactis *[55]. Recently, the use of *S. thermophilus *as a starter cultures for cottage cheese has gained popularity due to its faster acidification rate resulting in a faster production process in the dairy and a significant gain in capacity.

**Figure 2:**
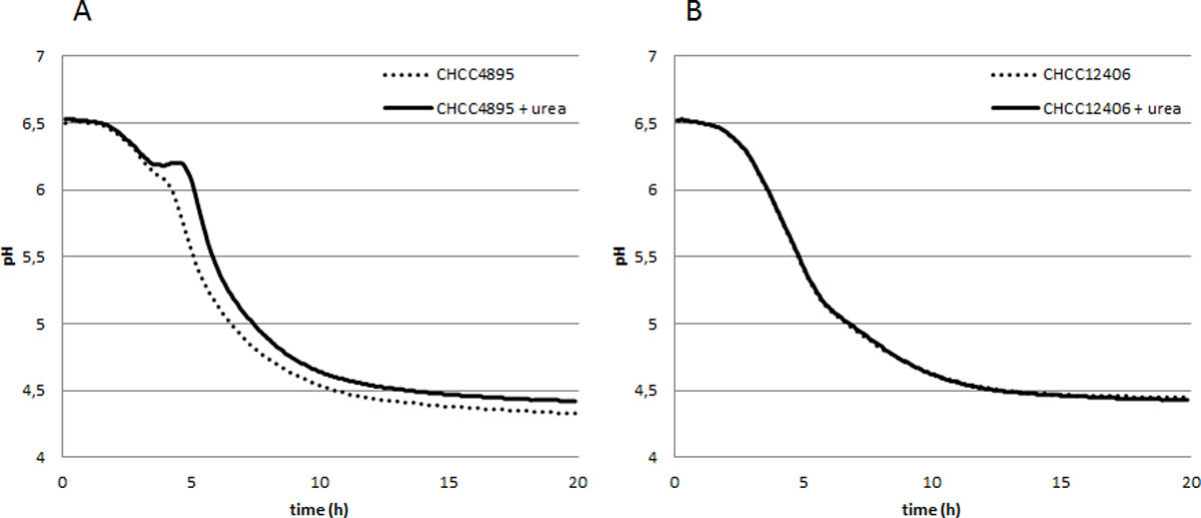
**Acidification curves (dashed line) of *S. thermophilus *CHCC4895 (A) and its urease mutant CHCC12046 (B) and the effect of the addition of urea (solid line)**.

**Figure 3:**
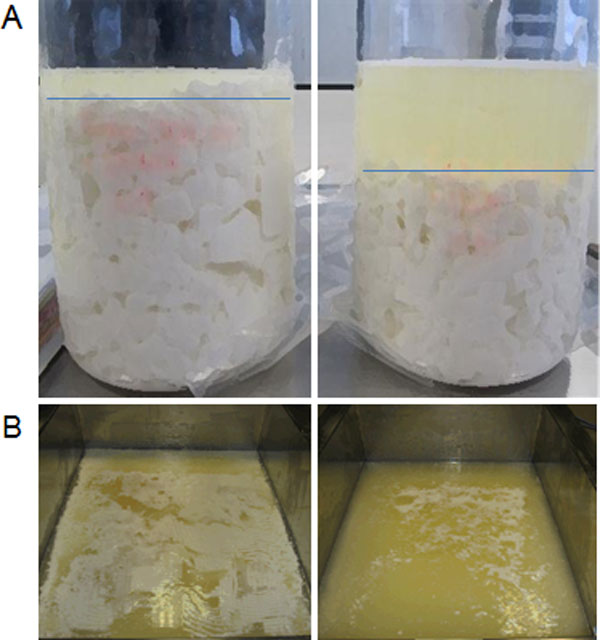
**Small scale cheese trails with strain CHCC4895 and its urease mutant CHCC12406**. Differences in the settlement of curd particles of both strains is illustrated in a 1-liter beaker (A) and small scale cheese vats (B). Left side strain CHCC4895; right side urease mutant CHCC12406.

*S. thermophilus *possesses amidohydrolase activity (urease; EC. 3.5.1.5) which converts urea into ammonia and carbon dioxide [56]. The carbon dioxide is incorporated into the cheese curd particles making them float which hinders whey removal and results in a loss of cheese mass. To tackle this problem a urease negative mutant of *S. thermophilus *CHCC4895 was isolated by random mutagenesis. Screening was done on plates containing a pH indicator; mutants devoid of urease activity lack buffering capacity due to reduced ammonia production. Mutant CHCC12406 did not show the typical pH increase when grown in milk with added urea, verifying the urease negative phenotype (Figure 2), and ammonia production was reduced by ca. 90%. Cheese trials made with CHCC12406 showed reduced floating of cheese curd particles (Figure 3) confirming the role of the *S. thermophilus *urease activity in the "floating curd" phenomenon and the possible use of cheese cultures based on urease negative mutants as a solution [57].

## Improving stress tolerance

Industrial production of starter cultures and their subsequent use in industrial processes for the production of fermented food subject the LABs to a large variety of stresses. For probiotic LABs, there is the additional stress associated with life in the human gastrointestinal tract. There is a strong interest in increasing the intrinsic tolerance of LABs to these stress factors.

### *Lactobacillus *strains with improved ethanol or bile tolerance

Malolactic fermentation mediated by LABs is important for flavor development in a number of wine varieties. Ethanol levels in wine have been increasing in recent years, possibly as a consequence of higher sugar levels in grapes caused by warmer growing seasons, putting stress on the malolactic bacteria. Consequently, development of malolactic bacteria that are resistant to higher levels of alcohol would be useful in the wine industry. Adaptation is often used to make microorganisms more tolerant to environmental stresses but this effect is only temporary. Resistance, on the other hand, is a permanent property which is typically acquired through genetic change. During the characterization of *Lb. plantarum *D-cycloserine resistant mutants, we discovered a number of unexpected phenotypes. For example, a number of the mutants are significantly more tolerant to ethanol than the parent strain. It was postulated that changes in the cell surface architecture result in reduced access of ethanol to the cell membrane thereby resulting in increased ethanol tolerance [58].

When efficacy of a probiotic strain is directly related to viability in the gastrointestinal tract, strain improvement can include increasing survival following exposure to gastric acid and bile. Multiple mechanisms are proposed to account for increased resistance to bile including efflux of bile acids/salts, modified sugar metabolism, and cell membrane or cell wall composition modification [59-61]. We also tested mutants of potentially probiotic *Lactobacillus *strains, selected using D-cycloserine resistance as described previously, for enhancement of bile tolerance. Some D-cycloserine resistant mutants showed higher survival rates than the parental strain when exposed to a bile shock and others demonstrated additional useful characteristics such as improved survival following gastric acid shock or desiccation. Genome sequence analysis showed unique mutations in each strain, though not all obviously related to D-cycloserine resistance. Importantly, for several highly bile tolerant isolates, there were no detectable changes in other *in vitro *characteristics used to evaluate probiotic potential, including adherence to mucus and stimulation of dendritic cell cytokine secretion.

### Acid production

The specific acids and the quantities produced by lactic acid bacteria in fermented products have major consequences on the properties of the final product. Acid production by LABs is therefore a desirable property to modulate and control.

### *Lactobacillus *strains with low post acidification

Strains of *Lb. delbrueckii *subsp*. bulgaricus *and *S. thermophilus *are essential culture components in the production of yoghurt. While many strains *of Lb. delbrueckii *subsp*. bulgaricus *display excellent texturizing properties, they might not be suitable for mild yoghurt applications due to the continued production of lactic acid during the shelf life of the yoghurt, a process referred to as "post-acidification". Current consumer preferences for yoghurt are for mild products with low post-acidification and a high texture.

A random mutagenesis and screening strategy was developed [62] to generate *Lb. delbrueckii *subsp*. bulgaricus *strains that combine excellent texturizing properties with low-post acidification. A strain with suitable texturizing properties but high post-acidification was treated with a mild mutagen and the resulting strain library screened in milk in microtiter plates using a colorimetric method for pH determination [4]. Mutants showing a higher end-pH than the mother strain were subsequently screened to find those which retained the desirable texturizing properties. This led to the isolation of a unique strain of *Lb. delbrueckii *subsp*. bulgaricus*, CHCC10019, which is able to acidify and texturize milk under industrially relevant conditions but is characterized by low post acidification. When using CHCC10019, the pH of the fermented milk drops by less than 0.20 pH units over 7 days at 8°C.

In another approach to obtain improved strains with reduced post-acidification the importance of the oligopeptide transport for growth fitness of *S. thermophilus *in milk was explored. The oligopeptide transport system in *S. thermophilus *consists of a seven proteins (AmiA1, AmiA2, AmiA3, AmiC, AmiD, AmiE and AmiF) and mutants exhibiting an altered oligopeptide transport system were found to have a reduced acidification rate [63]. The toxic oligopeptide analog aminopterine is transported into the cell by the Ami system. Accordingly, Garault *et al*. isolated Ami-deficient mutants with reduced post-acidification in milk by selecting for mutants that were resistant to this analog [64].

### Altering acidification properties by adapting carbohydrate metabolism

When grown in milk, the majority of *S. thermophilus *strains take up lactose and excrete galactose via the galactose-lactose antiporter [65]. In mozzarella cheese this excreted galactose can lead to "browning" upon heating of the cheese. This is ascribed to the Maillard reaction, where galactose, a reducing sugar, reacts with amino groups from amino acids. In addition, excess free galactose can lead to post acidification problems and imbalance in the cheese flora due to growth of resident lactic acid bacteria. Galactose positive wild type strains or galactose fermenting mutants are therefore interesting, especially for the purpose of reducing browning in pizza cheese.

**Figure 4:**
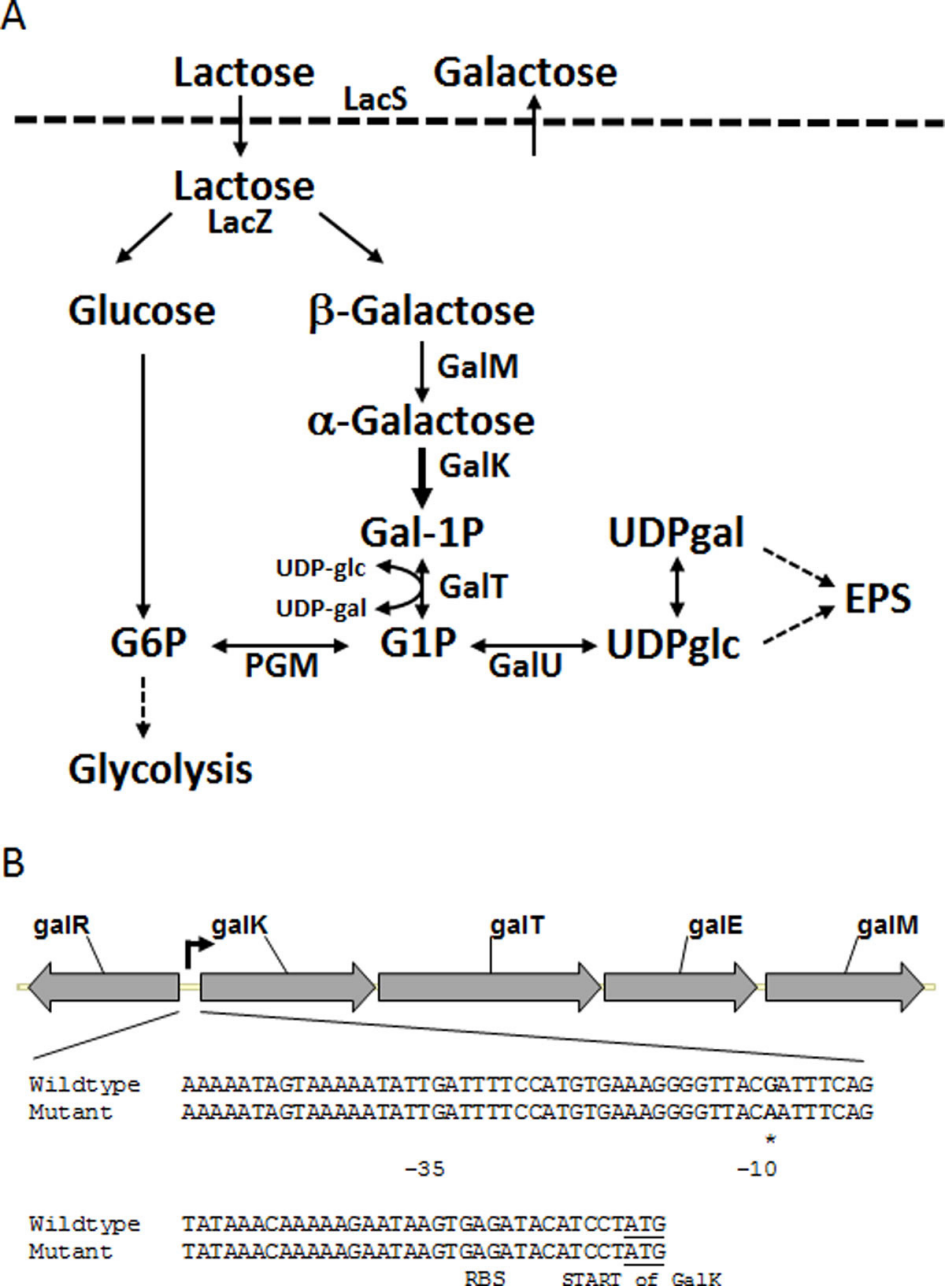
**Galactose and lactose metabolism in *S. thermophilus*, reproduced from Johansen *et al*. **[3]**(A)**. Abbreviations: LacS, lactose transporter; LacZ, β-galactosidase; GlcK, glucose kinase; GalM, mutarotase; GalK, galactokinase; PGM, α-phosphoglucomutase; GalT, galactose 1-phosphate udridyltransferase; GalE, UDP glucose 4-epimerase; GalU, UDP glucose pyrophosphorylase; gal1P, galactose-1-phosphate; g1p, glucose-1-phosphate; g6p, glucose-6-phosphate; UDP-glu, UDP-glucose; and UDP-gal, UDP-galactose. (B) The promoter region of the galactose operon; the mutation in the Pribnow box of the mutant strain in bold and marked (*). The enhanced expression of *galK *is indicated by a thickened arrow in the pathway and an arrow in the promoter region of the operon.

Most strains of *S. thermophilus *are unable to grow on galactose as sole carbon source; those that do grow use the Leloir pathway (Figure 4A). Nearly all strains contain the *gal *genes, but naturally occurring mutations in the *galK *promoter result in poor expression [66]. Screening 49 strains revealed only eight galactose fermenters [67] while we found 38 out of 247 *S. thermophilus *strains screened from our culture collection to have this capability. Since most strains do contain intact *gal *genes, it should be possible to isolate galactose positive variants from them. Mutants can be selected on M17 plates with galactose as sole carbon source and characterized by DNA sequencing. For example, the mutant mentioned in the section describing the optimization of the metabolic pathway of EPS has a mutation in the promoter region upstream of *galK *leading to increased expression of the *gal *genes. Similarly, strain CHCC14993, derived from the fast acidifying strain CHCC4323, has a G to A transition resulting in an improved Pribnow box 'TAC**A**AT' (Figure 4B). This single nucleotide exchange enables CHCC14993 to grow on galactose and results in an 11% (w/v) reduction in excreted galactose in milk fermented with this strain.

### Increasing the growth yield during fermentation

When LABs are grown in a batch culture, cells with the fastest growth rate dominate, often at the expense of biomass yield [68]. This is of concern to the starter culture industry as efficiency in the factory is dependent on the production of the maximum amount of biomass at the lowest cost [69]. Using an elegant emulsion system, it was possible to obtain *L. lactis *mutants with an increased growth yield [68] by eliminating the competitive advantage rapid growth gives in a liquid culture. The yield improvement occurred via mutations which divert the metabolism from lactate production towards mixed acid fermentation. Because these mutants cannot ferment lactose and produce reduced levels of lactate, they are not particularly useful for the dairy industry. They do, however, illustrate very nicely the tradeoff between growth rate and biomass yield that occurs in bacteria and suggest methods for changing the specific acids produced by a strain.

### *Lactobacillus *helveticus producing succinate

A variety of chemical analogues have been used to isolate mutants of *Lactobacillus *with improved flavor formation. One analogue, 3,4-dehydroproline, was expected to generate mutants with altered proline and glutamate metabolism in *Lb. helveticus*. However, when the mutants were characterized, one mutant showed a biphasic acidification of milk and was found to be unable to produce succinate. Genome sequencing revealed a single mutation; it was in an L-lactate dehydrogenase (EC 1.1.1.27; lhe_1813). This finding is consistent with the deviation in acidification pattern but does not readily explain the lacking succinate production. Perhaps the L-lactate dehydrogenase also serves as a malate dehydrogenase. A strain defective in malate dehydrogenase will not produce fumarate and consequently no succinate. However, the isolation of a lactate/malate dehydrogenase mutant using 3,4-dehydroproline was not expected and cannot be readily explained. This illustrates an added benefit of direct selection methods and the use of analogs: surprising observations might lead to new insight into the metabolism of LABs.

## Summary and future perspectives

There is a constant need for new strains for inclusion in starter cultures for the food fermentation industry. New strains can either come from screening of wild-type strains from natural sources as described by Johansen *et al*. [4] or improvement of existing strains as described herein. Many industrially relevant properties can be addressed by these techniques. These include: enhancement of bacteriophage resistance; improvement of the texturizing capability of strains for yoghurt production; elimination of traits undesirable in any food fermentation; elimination of traits undesirable in specific food fermentations; improvement of stress tolerance; and modulation of both the specific acids produced during fermentation as well as the amounts produced. Classical strain improvement can be combined with natural methods of gene transfer like conjugation, transduction and natural transformation to create industrial strains with improved properties in a variety of genetic backgrounds.

An alternative approach is strain improvement using established techniques of recombinant DNA technology. A large number of methods have been developed to create genetically modified organisms which fulfil a published definition of 'food-grade' [69,70]. These are not currently used for the direct development of industrial starter cultures due to concerns over consumer acceptance and regulatory approval. Recombinant DNA technology is, however, routinely used in research laboratories for proof-of-concept and subsequently followed up by the development of similar strains created through the use of classical strain improvement. The advantages and disadvantages of the various methods of strain improvement are described in Table 1.

Recent advances in the field of genome editing may provide additional ways of improving strain performance. Technologies like recombineering [71] and use of the CRISPR-Cas9 system [72] allow for precise modification, at the single nucleotide level, in a genome of interest. This allows the production of strains which will be totally indistinguishable from strains produced by classical strain improvement. This high level of specificity in genome editing will require a reassessment of not only the definition of a genetically modified organism but also the reasons behind discriminating strains based on the methods used to develop them.

## List of abbreviations used

Abi: abortive infection; CPS: Capsular polysaccharide; EPS: Extracellular polysaccharide; GMO: Genetically modified organism; IS: Insertion sequence; *L.: Lactococcus; *LAB: Lactic acid bacteria; *Lb.: Lactobacillus; O.: Oenococcus; *PIP: Phage infection protein; *S.: Streptococcus*.

## Competing interests

All authors are employed by Chr. Hansen A/S.
